# Psychiatric aspects of the end of life in oncologic patients

**DOI:** 10.1192/j.eurpsy.2021.1982

**Published:** 2021-08-13

**Authors:** M. Trigo

**Affiliations:** Psychiatry, Centro Hospitalar Universitário do Algarve, Faro, Portugal

**Keywords:** Antidepressants, oncology, psychiatry, end of life

## Abstract

**Introduction:**

Patients with life-limiting oncologic conditions should be approached by multidisciplinary teams that contribute to improve their quality of life, including support from mental health dedicated professionals. It is the role of the psychiatrist to understand the relationship between mental health and general health outcomes, specific of this type of patients. Terminally ill and dying patients benefit from psychiatric support, and it seems to have real effects in terms of patient care and medical staff education.

**Objectives:**

To identify approaches and mental health professionals’ practices regarding end-of-life issues in terminally ill cancer patients.

**Methods:**

Review of the most recent literature regarding end-of-life issues in terminally ill cancer patients. The research was carried out through the Cochrane, UptoDate, PubMed, MedLine, LILACS and SciELO databases, using the terms “oncology”, “psychiatry” and “end of life”, until December 2020.

**Results:**

While symptoms of anxiety and depression are common in palliative care settings, generally related to feelings of helplessness and fear of death, they should not be assumed to be an inevitable part of it. For terminally ill patients, anxiety and trauma-related disorders can manifest in various ways and it is important to establish personalized treatment approaches, based on a supportive clinical team, and, if necessary, psychotherapy and psychopharmacologic or complementary treatments.

**Conclusions:**

It is extremely important to assess terminally ill patients from the mental health point of view. It is required that psychiatrists take part in clinical care and research on the treatment of these patients with severe medical conditions, in order to increase their quality of life.

**Disclosure:**

No significant relationships.

